# ‘More than a feeling’: An empirical investigation of hedonistic accounts of animal welfare

**DOI:** 10.1371/journal.pone.0193864

**Published:** 2018-03-12

**Authors:** Jesse Robbins, Becca Franks, Marina A. G. von Keyserlingk

**Affiliations:** 1 Animal Welfare Program, Faculty of Land and Food Systems, University of British Columbia, Vancouver, British Columbia, Canada; 2 Department of Environmental Studies, New York University, New York City, New York, United States of America; Universidade do Porto Instituto de Biologia Molecular e Celular, PORTUGAL

## Abstract

Many scientists studying animal welfare appear to hold a hedonistic concept of welfare -whereby welfare is ultimately reducible to an animal’s subjective experience. The substantial advances in assessing animal’s subjective experience have enabled us to take a step back to consider whether such indicators are all one needs to know if one is interested in the welfare of an individual. To investigate this claim, we randomly assigned participants (*n* = 502) to read one of four vignettes describing a hypothetical chimpanzee and asked them to make judgments about the animal’s welfare. Vignettes were designed to systematically manipulate the descriptive mental states the chimpanzee was described as experiencing: feels good (FG) vs. feels bad (FB); as well as non-subjective features of the animal’s life: natural living and physical healthy (NH) vs. unnatural life and physically unhealthy (UU); creating a fully-crossed 2 (subjective experience) X 2 (objective life value) experimental design. Multiple regression analysis showed welfare judgments depended on the objective features of the animal’s life more than they did on how the animal was feeling: a chimpanzee living a natural life with negative emotions was rated as having better welfare than a chimpanzee living an unnatural life with positive emotions. We also found that the supposedly more purely psychological concept of happiness was also influenced by normative judgments about the animal’s life. For chimpanzees with positive emotions, those living a more natural life were rated as happier than those living an unnatural life. Insofar as analyses of animal welfare are assumed to be reflective of folk intuitions, these findings raise questions about a strict hedonistic account of animal welfare. More generally, this research demonstrates the potential utility of using empirical methods to address conceptual problems in animal welfare and ethics.

## Introduction

Various lines of evidence suggest that concern about the welfare of animals is increasing [1]. In response, the field of animal welfare emerged to provide empirical data on how different factors influence animal welfare. Scientists working in this field typically draw inferences about welfare based on changes in behavior and physiological functioning. Although still a relatively new field, the number of scientific papers addressing animal welfare has increased significantly in the last 20 years [2]. Much of this research is used to inform legislative and regulatory policies, as well as private accreditation schemes [3].

However, drawing inferences about animal welfare presupposes some conception of what welfare is [4], and there is still no universally agreed upon definition of welfare [5, 6]. Yet among scientists studying animal welfare, there appears to be strong support for one particular theory known as welfare hedonism (Table 1). Welfare hedonism is the view that subjective experience is the only non-instrumentally valuable constituent of welfare [7]. On this view, an animal’s welfare is diminished if, and only if; the animal experiences negatively-valenced feelings (referred to generally as ‘pain’, but also including various other negative emotions such e.g. “fear”, “depression”, “boredom”) and enhanced if, and only if, the animal experiences positively-valenced feelings (referred to generally as ‘pleasure’) [8, 9]. Some have gone even further and suggested that life-focused concepts such as ‘quality of life’ and “a life worth living” also refer to the net balance of positive over negative psychological states albeit over a more extended period of time [10–14].

**Table 1:** Select quotations from scientists studying animal welfare representing welfare hedonism.

“…animal welfare is dependent solely on the mental, psychological and cognitive needs of the animals concerned…as long as the mental state is protected (i.e., as long as the animal “feels” all right) then its welfare will be all right…animal welfare is dependent solely on the cognitive needs of the animals concerned”	[15]
“…something can only affect the welfare of an animal if it affects the conscious experiences of the individual.”	[9]
“…the animals perception of its condition must serve as the basis for well-being…”	[16]
“Animal welfare consists of the animal’s positive and negative experiences.”	[17]
“…welfare will depend on the relative preponderance of positive over negative experiences during the animal’s lifetime.”	[10]
“Quality of life refers to a state of mind; it is conscious, subjective, mental experience.”	[11]
“Welfare is a characteristic of animals, i.e. it is a descriptive property of animals…The welfare state of an animal is determined by all the emotional states and only the emotional states insofar as they are experienced subjectively by that animal….Per definition, a drugged animal that is kept in a permanently euphoric state has high welfare status even though it may be questioned whether this is morally acceptable.”	[18]
“Welfare is fulfilled when the animals do not feel any long lasting negative emotions and when they can experience positive emotions.”	[19]
“…animal welfare is all to do with the secondary, subjective feelings, with the absence of negative feelings, particularly the strong negative feelings we call suffering and with the presence of positive feelings that we call pleasure.”	[8]
“An individual’s overall welfare depends on the combination of all its current experiences…Like overall welfare, Quality of Life is a matter of the animal’s mental experiences. It is effectively a balance of all experiences within a specific period.”	[12]
“Animal welfare is a state within the animal…how the animal feels now.”	[20]
“Animal welfare is a state that is subjectively experienced by an animal; it is a state within the animal.”	[21]
“The welfare of any sentient animal is determined by its individual perception of its own physical and emotional state.”	[22]
“Welfare is net happiness (enjoyment minus suffering).”	[23]

These hedonistic theories of welfare construe welfare as a descriptive concept [24]; whereby, determining whether or not an animal has good welfare is solely a matter of accurately representing or describing the animal’s mental states. If the animal meets the requisite psychological criteria (i.e. low negative affects and high positive affect) then the concept is said to apply and the animal has good welfare. Normative judgments regarding other potentially welfare-relevant aspects, of the sort commonly associated with objective list theories (e.g. physical health, naturalness, bodily integrity) are viewed as only valuable insofar as they result in changes in the animal’s subjective experience.

We believe there is good reason to question welfare hedonism. It is plausible that welfare is not reducible to experiencing certain mental states, because the concept is not purely descriptive. This alternative view posits welfare as a thick concept that does not function to merely represent or describe an animal’s mental states, but also to evaluate their broader life circumstances more generally [25]. Having good welfare means more than feeling great all the time, it entails living a life we endorse, encourage or recommend to others [26], and lives consist of more than just facts about mental states of the subject that occupies them.

Especially for a publicly ‘mandated’ science such as animal welfare [6], one way to help adjudicate these competing theoretical possibilities is to determine which of them best reflects ordinary, common sense usage [27, 28]. The field of experimental philosophy is based on the idea that by investigating patterns in how ordinary people apply (or do not apply) their concepts in particular situations we can contribute to certain types of conceptual analyses [29]. Applied to animal welfare, this approach resonates with calls to adopt a scientific conception of welfare that corresponds to its everyday meaning [30]. The extent to which scientists working on animal welfare expect their work to address public concerns about this topic, namely assessing where the public stands, is directly relevant to the scientific community.

We thus set out to provide a preliminary study of the folk concept of animal welfare. Contrary to popular hedonistic conceptions of animal welfare put forth by many scientists, we hypothesized that judgments of animal welfare would be influenced by factors other than the animal’s subjective experience. We predicted that normative judgments about the life the animal was living would influence welfare judgments. In the strong version of our hypothesis, these alternative pieces of information would play an even greater role in welfare assessments than information about the mental state of the individual animal.

## Methods

This study received ethics approval from the Behavioural Research Ethics Board (H15-03053) at the University of British Columbia.

Participants (n = 502) were recruited using Amazon Mechanical Turk. To limit concerns about self-selection bias the recruitment advertisement simply read, “give us your opinions.” Restrictions were set to limit the survey to US residents with a requester approval rating of 95% or greater.

Participants were randomly assigned to read one of four vignettes describing the life of a hypothetical chimpanzee. Prior to receiving their vignette they were told that the scenarios they were about to read were not necessarily realistic and they should “suspend disbelief” and “imagine” that what they read was true—a technique that is common in philosophical thought experiments [31]. Following the basic principles of the Contrastive Vignette Technique [32], scenarios were designed to systematically manipulate key variables of interest while keeping all other aspects of the vignettes as similar as possible (Table 2). The vignettes manipulated the descriptive mental states the chimpanzee was described as experiencing: feels good (FG) vs. feels bad (FB); as well as the normative value of her life: natural and physically healthy (NH) vs. unnatural and physically unhealthy (UU) creating a fully crossed 2 (subjective experience) x 2 (objective life value) experimental design (Table 2). After reading their vignette, participants answered three questions assessing their level of agreement/disagreement with the following statements: “Sally is happy”, “Sally has a life worth living” and “Sally is unhappy” and three more asking them to describe Sally’s “welfare”, “well-being” and “quality of life” on a scale ranging from extremely bad to extremely good. All questions used 7-point likert-type response scales. To assist in identifying subjects that may have been unable to suspend disbelief (aka “unconscious realists”) we included a manipulation check asking them whether or not they believed, “Sally spends all of her time feeling excellent [terrible]”. At the end of the study participants answered basic demographic questions, were thanked, debriefed about the study’s aims and compensated ($0.50).

**Table 2:** Vignettes for each experimental condition.

*Feels good/natural life*, *physically healthy*. Sally is a female chimpanzee living in the jungle. She lives in a troop with six other chimpanzees and often interacts with them. She spends most of her days roaming the jungle and foraging for food. She is in good physical health and has many healthy offspring who also live with her. Along with her food, Sally eats Aspilia leaves every day. These leaves serve as a natural stimulant that promotes mental health. A team of neuropsychologists and primate experts, using state-of-the-art technology, has recently determined that Sally spends almost all of her time feeling excellent.	*Feels bad/ natural life*, *physically healthy*. Sally is a female chimpanzee living in the jungle. She lives in a troop with six other chimpanzees and often interacts with them. She spends most of her days roaming the jungle and foraging for food. She is in good physical health and has many healthy offspring who also live with her. Along with her food, Sally eats Aspilia leaves every day. These leaves serve as a natural stimulant that promotes mental health. Nonetheless, a team of neuropsychologists and primate experts, using state-of-the-art technology, have recently determined that Sally spends almost all of her time feeling terrible.
*Feels good/unnatural life physically unhealthy*. Sally is a female chimpanzee living in a primate research facility. She lives alone apart from any other chimpanzees and seldom interacts with her caretakers. She spends most of her days in her indoor enclosure, waiting for food to be delivered. She is in poor physical health and has never had any offspring. Along with her food, Sally is given a dose of psychoactive drugs every day. These drugs serve as an artificial stimulant that promotes mental health. A team of neuropsychologists and primate experts, using state-of-the-art technology, has recently determined that Sally spends almost all of her time feeling excellent.	*Feels bad/ unnatural life physically unhealthy*. Sally is a female chimpanzee living in a primate research facility. She lives alone apart from any other chimpanzees and seldom interacts with her caretakers. She spends most of her days in her indoor enclosure, waiting for food to be delivered. She is in poor physical health and has never had any offspring. Along with her food, Sally is given a dose of psychoactive drugs every day. These drugs serve as an artificial stimulant that promotes mental health. Nonetheless, a team of neuropsychologists and primate experts, using state-of-the-art technology, have recently determined that Sally spends almost all of her time feeling terrible.

Prior to data collection it was decided that subjects would be excluded from analysis for any one of the following reasons: a) missing responses, b) indicating that English was not their native language, c) having an IP address originating outside the US, or d) failing the manipulation check (reported that they could not believe our description of Sally’s mental states).

## Statistical analyses

A multiple regression approach was used to model welfare and well-being judgments. In separate regression models, we used the participant’s answer to target questions (e.g. happiness, quality of life, etc.) as the outcome and the “feels good-feels bad” factor, the “natural/healthy-unnatural/unhealthy” factor, and their interaction as predictors. This approach is mathematically identical to an ANOVA approach, but reports an intercept and t-tests for individual slopes as the output. In a multiple regression, the output reflects the underlying linear model, with an intercept and slopes comparing different conditions.

In our specific model set up, the intercept represented the model prediction for the outcome variable (e.g. mean happiness) for a participant in the FB/UU condition and the slopes tests for the significance of a particular effect. Accordingly, the “Feels good” slope represents the statistical difference between the FB/UU condition and the FG/UU condition; the “Natural/healthy” slope represents the statistical difference between the FB/UU condition and the FB/NH condition; the “Feels good x NH” slope represents the statistical difference of the interaction between the two factors.

## Results

After data exclusions (*n* = 29) our final sample consisted of 473 participants (51% female, Age: *M* = 38, *SD* = 12).

**Fig 1:**
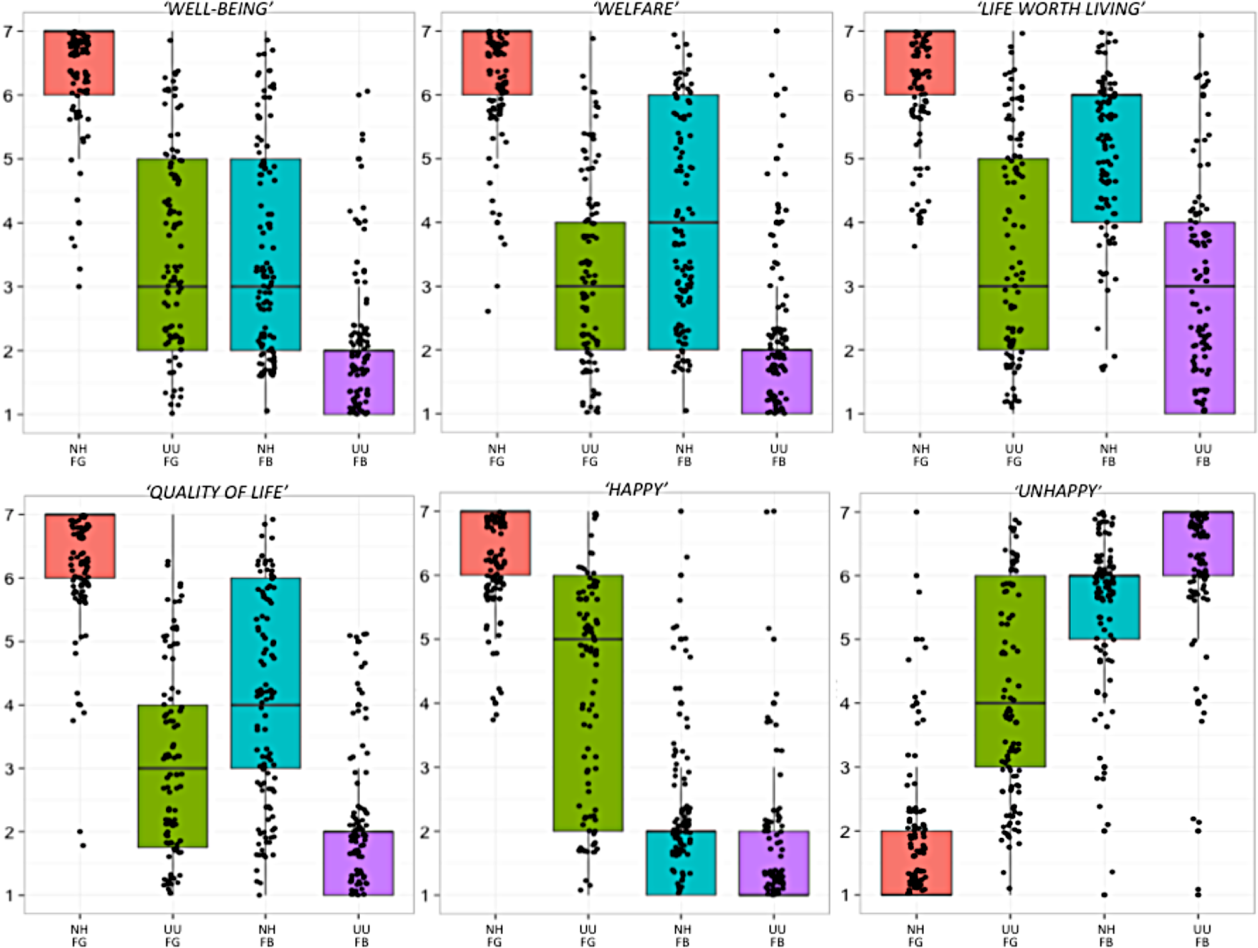
Participant ratings (*n* = 502) for each concept across all four conditions: Natural life/healthy and feels good (NH-FG); unnatural life/unhealthy and feels good (UU-FG); natural life/healthy and feels bad (NH-FB) and unnatural life/unhealthy and feels bad (UU-FB). For the statements: "Sally is happy”, "Sally is unhappy" and "Sally has a life worth living” participants indicated their agreement using a 7-point likert-type scale (1 = strongly disagree, 7 = strongly agree). Participants were asked to describe Sally's well-being, welfare and quality of life using a 7-point scale (1 = extremely bad, 7 = extremely good).

As expected, we found that subjects generally tended to assess welfare (and related concepts) as being higher with greater positive emotions (FG) and when the chimpanzee was living in more natural living conditions and biologically healthy (NH), but these effects were moderated for all outcome variables (Table 3, Fig 1). Inspecting the nature of the interactions more carefully revealed several patterns. First, for all outcome variables, we found that the effect of subjective feelings was smaller when the chimpanzee was living an unnatural and unhealthy life versus a natural and healthy life. Second, we found that these features (i.e. living conditions and physical health) had a greater effect than feelings on participant’s judgments of several welfare-related concepts: “welfare” (FB-NH: 4.02 vs. FG-UU: 2.94, t(247) = 5.17, p < 0.0001), “well-being” (FB-NH: 3.70 vs. FG-UU: 3.24, t(247) = 2.16, p < 0.05) “quality of life” (FB-NH: 4.08 vs. FG-UU: 2.76, t(247) = 6.29, p < 0.0001), and “life worth living” (FB-NH: 5.32 vs. FG-UU: 3.46, t(247) = 9.12, p < 0.0001).

**Table 3:** Effect of treatment: Feels good (FG) vs. feels bad (FB) and natural living and physical healthy (NH) vs. unnatural life and physically unhealthy (UU) for each concept tested including interaction term.

*Concept*	*β*	*SE*	*t-value*	*P*
HAPPY				
Intercept	1.46	0.12		
Feels good (*vs* Feels bad)	2.54	0.17	15.30	<.0001
NH (*vs* UU)	0.72	0.16	4.39	<.0001
Feels good x NH	1.64	0.23	7.04	<.0001
UNHAPPY				
Intercept	6.35	0.12		
Feels good (*vs* Feels bad)	-2.23	0.18	-12.56	<.0001
NH (*vs* UU)	-0.68	0.18	-3.84	.0001
Feels good x NH	-1.72	0.25	-6.90	<.0001
WELFARE				
Intercept	2.11	0.13		
Feels good (*vs* Feels bad)	0.92	0.18	5.00	<.0001
NH (*vs* UU)	1.83	0.18	10.06	<.0001
Feels good x NH	1.57	0.26	6.08	<.0001
WELL-BEING				
Intercept	1.83	0.12		
Feels good (*vs* Feels bad)	1.56	0.18	8.88	<.0001
NH (*vs* UU)	1.75	0.17	10.07	<.0001
Feels good x NH	1.43	0.25	5.81	<.0001
QUALITY OF LIFE				
Intercept	1.88	0.12		
Feels good (*vs* Feels bad)	0.99	0.18	5.54	<.0001
NH (*vs* UU)	2.13	0.18	11.98	<.0001
Feels good x NH	1.44	0.25	5.74	<.0001
LIFE WORTH LIVING				
Intercept	2.95	0.14		
Feels good (*vs* Feels bad)	0.60	0.20	6.68	.003
NH (*vs* UU)	2.33	0.20	11.95	<.0001
Feels good x NH	0.46	0.23	5.15	.10

## Discussion

Our results provide some preliminary support for the notion that subjective experience may not be the whole story when people assess animal welfare and welfare-related concepts. They generally replicate and extend previous research showing that the concept of happiness appears not to be restricted to describing mental states. For example, a cross-cultural study of historical trends of language use found that happiness has most commonly been equated with favorable external conditions and not internal feelings [33]. An extensive series of experimental studies [34–36] found judgments of happiness were heavily influenced by normative evaluations about the life the person was described as living and not simply the mental states they were experiencing. Taken together with our results, these findings lend some support to the conjecture that the concept of happiness shares functional similarities when applied to some non-human animals [27]. We suggest that further theoretical insights into animal welfare might be found by attending more closely to the much more extensive philosophical and scientific literatures on human welfare [37, 38].

Our life value manipulation emphasized two frequently encountered non-subjective aspects of welfare—physical health and natural living. While these features are typically associated with objective list theories of welfare, some theorists include additional factors as well [39]. Our goal in this study was not to independently evaluate among all the potential objective list items, but rather to test the predictions of hedonism.

Our results also do not preclude the possibility that other, non-hedonic mental state accounts of welfare may prove to be more aligned with the ordinary understanding (e.g., desire theories) [40, 41]. More research is needed to test folk intuitions regarding these non-hedonic models of welfare.

Although our results support to the view that the concept of welfare functions similarly for both non-humans and humans alike, this effect seems likely to differ across species. While it is generally the case that moral concern for animals is growing, the strength of these concerns varies considerably between species [42]. Some studies have shown these concerns increase proportionally with perceived biological and behavior similarity with humans [43], while other work paints a more complex picture whereby factors such as emotional attachment to individual animals as well as historical and cultural influences mediate this relationship [44]. Including covariates that take these variations in concern towards different animals into account might provide a more nuanced picture of the role they play in shaping and influence judgments of animal welfare.

Interestingly, the interaction between the animal’s feelings and life value indicated that the influence of life value was less pronounced when the chimpanzee was described as feeling bad as opposed to when she was described as feeling good. The finding that judgments of unhappiness were less influenced by evaluative considerations is consistent with previous research showing the presence of negative feelings did in fact largely explain judgments of unhappiness, but not happiness [34]. This asymmetry between the seemingly polar opposite concepts of happiness and unhappiness could have implications for the field of animal welfare. As the field shifts its focus from the prevention of negative welfare to the promotion of positive welfare [45, 46] the role of evaluative judgments may become relatively more prominent.

Our methodological approach offers several potential advantages over previous research examining how different groups conceptualize animal welfare. Unlike previous work [47–50], our experimental design allowed us to systematically manipulate key variables in order to test a specific hypothesis. Furthermore, instead of directly asking people to define animal welfare (e.g. “What does good animal welfare mean?”), we inferred their understanding of welfare based on how they used the concept. This indirect approach to studying concepts respects the fact that people can use a concept while being unable to express information about its properties [51].

A notable limitation of this study is our inability to be certain that our manipulations of subjective experience were perceived identically. Our inclusion of a manipulation check was designed to address this issue. This strategy has been utilized in other empirical work addressing complex philosophical problems [52], but it still cannot guarantee that pleasure was perceived as quantitatively identical. We hope future research will devise better ways of understanding how the often-unrealistic assumptions involved in many philosophical thought experiments are interpreted by participants and how this might possibly bias results.

We began this paper by suggesting that many scientists studying animal welfare endorse welfare hedonism. This is currently just an assumption based on the writings of scientists working in this field and our own personal experiences. The only study involving animal welfare experts we are aware of reported that scientists tended to equate welfare with subjective feelings, whereas, lay conceptions included additional factors [48]. We hope future research will explore the views of scientists working in animal welfare so we can speak more definitively about how closely they reflect (or not) those of ordinary people.

At a more general level, our findings support the classification of happiness and its cognates as ‘thick concepts’, which have the dual function of simultaneously describing and evaluating [26]. When people are deciding whether or not an animal has good welfare they appear not only to be trying to determine whether some factual state of affairs obtains (i.e. whether or not the animal is experiencing certain emotions), but are also making a more general evaluation about the life the animal is living. The idea that life evaluation is an important aspect of judgments of welfare has been explored in detail [53, 54] for humans, but much less attention has been paid in case of non-human animals. We hope that this research will inspire more conversation about this interesting possibility.

Viewing animal welfare as a thick concept affected by factors beyond how the animal feels might explain some of the skepticism expressed towards some animal welfare research [55]. If one is of the opinion that some forms of animal use are morally illegitimate, perhaps because animals are being used as mere means, then the very notion that they can have good welfare in such circumstances may strike many as odd. The thick conception of welfare suggests that moral views about the legitimacy of different human-animal relations should alter judgments about whether or not an animal is faring well. Contrary to the pure science model of animal welfare, which argues that welfare can be scientifically studied without invoking any value judgments [56], the thick conception of welfare supports Tannenbaum’s contention that, “it is impossible to use the term welfare as it is ordinarily employed by people without committing oneself to certain ethical judgments.” [57]. At a minimum, however, those of us working in this field should generate necessary data points for the unfolding conversation of what welfare is.

Along with other scientists [58–60], we believe that merging the gap between science and public perception is a central project that must be given attention. While it is not necessary for scientists working in this field to simply assume the infallibility of public perceptions, it is important for them to understand how their work is likely to be perceived by non-specialists. Additionally, it is possible that with research such as ours, new and future insights may aid in explaining to the public why alternative perspectives on welfare are necessary and/or valid. Thus, we hope that work such as ours can help contribute to the ongoing dialogue between science and society.

## Conclusion

It has previously has been suggested that the study of animal welfare and ethics will benefit from experimental philosophy research [61]. Here we have described an attempt to experimentally examine whether the folk concept of animal welfare is consistent with the predictions of strict hedonism. We found evidence that folk judgments of animal welfare were not fully determined by the animal’s subjective experience. Welfare attributions were influenced by normative considerations about the life the animal was living. It appears that it may be possible to distinguish how an animal is feeling from how their life is going.
